# Tuberculous Lymphadenitis in Ethiopia Predominantly Caused by Strains Belonging to the Delhi/CAS Lineage and Newly Identified Ethiopian Clades of the *Mycobacterium tuberculosis* Complex

**DOI:** 10.1371/journal.pone.0137865

**Published:** 2015-09-16

**Authors:** Fantahun Biadglegne, Matthias Merker, Ulrich Sack, Arne C. Rodloff, Stefan Niemann

**Affiliations:** 1 College of Medicine and Health Sciences, Bahir Dar University, Bahir Dar, Ethiopia; 2 Institute of Medical Microbiology and Epidemiology of Infectious Diseases, University Hospital, University of Leipzig, Leipzig, Germany; 3 Institute of Clinical Immunology, University Hospital, University of Leipzig, Leipzig, Germany; 4 Molecular Mycobacteriology, Research Center Borstel, Borstel, Germany; 5 Translational Centre for Regenerative Medicine (TRM)-Leipzig, University of Leipzig, Leipzig, Germany; 6 German Center for Infection Research, Partner Site Borstel, Borstel, Germany; St. Petersburg Pasteur Institute, RUSSIAN FEDERATION

## Abstract

**Background:**

Recently, newly defined clades of *Mycobacterium tuberculosis* complex (MTBC) strains, namely Ethiopia 1–3 and Ethiopia H37Rv-like strains, and other clades associated with pulmonary TB (PTB) were identified in Ethiopia. In this study, we investigated whether these new strain types exhibit an increased ability to cause TB lymphadenitis (TBLN) and raised the question, if particular MTBC strains derived from TBLN patients in northern Ethiopia are genetically adapted to their local hosts and/or to the TBLN.

**Methods:**

Genotyping of 196 MTBC strains isolated from TBLN patients was performed by spoligotyping and 24-loci mycobacterial interspersed repetitive unit-variable number of tandem repeats (MIRU-VNTR) typing. A statistical analysis was carried out to see possible associations between patient characteristics and phylogenetic MTBC strain classification.

**Results:**

Among 196 isolates, the majority of strains belonged to the Delhi/CAS (38.8%) lineage, followed by Ethiopia 1 (9.7%), Ethiopia 3 (8.7%), Ethiopia H37RV-like (8.2%), Ethiopia 2 and Haarlem (7.7% each), URAL (3.6%), Uganda l and LAM (2% each), S-type (1.5%), X-type (1%), and 0.5% isolates of TUR, EAI, and Beijing genotype, respectively. Overall, 15 strains (7.7%) could not be allocated to a previously described phylogenetic lineage. The distribution of MTBC lineages is similar to that found in studies of PTB samples. The cluster rate (35%) in this study is significantly lower (P = 0.035) compared to 45% in the study of PTB in northwestern Ethiopia.

**Conclusion:**

In the studied area, lymph node samples are dominated by Dehli/CAS genotype strains and strains of largely not yet defined clades based on MIRU-VNTR 24-loci nomenclature. We found no indication that strains of particular genotypes are specifically associated with TBLN. However, a detailed analysis of specific genetic variants of the locally contained Ethiopian clades by whole genome sequencing may reveal new insights into the host-pathogen co-evolution and specific features that are related to the local host immune system.

## Background

Tuberculosis (TB) remains a major global health problem in Ethiopia, regardless of having highly efficacious treatment for decades [1]. According to the World Health Organization (WHO) global TB report in 2013, Ethiopia has been one of the highest TB burden countries with an incidence rate of 261 cases per 100,000 populations in 2012 [1]. Extra-pulmonary TB (EPTB) contributes to the problem. TB lymphadenitis (TBLN) is the most common form of EPTB and accounts for 80% of all new EPTB cases in Ethiopia [2]. The TB problem in Ethiopia is deteriorating with the emergence and spread of drug-resistant TB strains [3, 4]. Indeed, Ethiopia has one of the highest incidence rates worldwide with more than 5000 estimated MDR-TB patients each year [5]. The WHO report in 2013 showed that the prevalence of MDR-TB has been increasing in newly diagnosed and previously treated TB patients [1], indicating TB is a major public health problem in Ethiopia.

Mycobacterial species culture is not available as a routine TB diagnostic method in Ethiopia [6, 7]. Thus, a laboratory investigation of TB in Ethiopia is mainly done on smear microscopy, known to exhibit a lower sensitivity and specificity compared to culture based methods, e.g. MGIT, LJ [6–10]. Furthermore, a lack of species identification and drug susceptibility testing (DST) for this method is another major problem in terms of diagnostic capacities and TB surveillance [7]. Studies have shown that genotyping of TB are key factors in the control of TB [11–17], by helping to identify sources of infection, TB patients who are involved in recent transmission and reactivation of old infection. However, the utility of these methods is limited in resource poor countries like Ethiopia, where TB rates are high. In Ethiopia, only limited data is available on the association of particular *Mycobacterium tuberculosis* complex (MTBC) strains and their ability to disseminate in other tissues of the body. Recently, four newly defined clades of TB strains associated with active pulmonary TB (PTB) in up to one-third of the patients, namely Ethiopia 1–3 and Ethiopia H37Rv-like strains, were identified in northwestern Ethiopia [17]. Another study analyzing the distribution of genotypes among PTB and TBLN patients in Ethiopia, reported a similar distribution of identified genotypes between the two manifestations of the disease [16]; however, the limitation of this study was that highly discriminatory MIRU-VNTR typing of 24 loci was only available for a subset of strains. Additionally, using whole genome sequencing of selected isolates with an unusual spoligotype pattern, the authors confirmed the presence of a new MTBC lineage, namely lineage 7 Ethiopia.

In this study, we applied 24-loci MIRU-VNTR typing and spoligotyping for the entire strain collection to assess the presence of recently, newly defined lineage 7 and link the newly collected data to the definition of the new lineages Ethiopia 1–3 and Ethiopia H37Rv-like strains. We further analyzed whether these new MIRU-VNTR-based subtypes differ in their ability to cause lymphadenitis and explored the question if these strain types in northern Ethiopia are genetically adapted to their local hosts and/or to the TBLN. With this background, this study was conducted to extend our understanding of the diversity; phylogeny, and transmission dynamics of MTBC strains isolated from TBLN patients.

## Material and Methods

### Study population, specimen collection, storage and transport

All TBLN patients diagnosed between April and May 2012 (n = 226) at four main hospitals (Felege Hiwot, Gamby, Gondar, and Dessie) and at Bikat diagnostic clinic in northern Ethiopia were included in the study. The fine needle aspirate (FNA) samples were collected from lymph nodes of all patients and were divided into two halves one for cytology and the other for culture. The diagnoses of TBLN using fine needle aspiration cytology have been clearly defined [18]. A structured and pretested questionnaire was used to collect demographic characteristics of study subjects. The specimens were stored and transported to the Institute of Medical Microbiology and Epidemiology of Infectious Disease, University Hospital in Leipzig, Germany as described previously [7].

### Mycobacterium culture, drug susceptibility testing (DST), and DNA extraction

Specimens were processed for culture and DST as described previously [7, 19, 20]. Briefly, 10 ml of 0.5% NALC solution (4% NaOH and 2.9% sodium citrate) was added to each aspirate sample. Then the specimens were incubated at room temperature on a shaker for 20 minutes, after which 30ml of phosphate buffered saline (PBS) (pH 6.8) was added for neutralization and the specimens were subsequently centrifuged at 3,300xg for 20 minutes. The concentrated specimens were re-suspended in 1ml of phosphate buffer and used to perform mycobacterial culture. *Mycobacterium* species identification was carried out using the DNA hybridization technique (Genotype MTBC, Hain Life sciences, Nehren, Germany). For DNA extraction, 1ml of liquid culture was transferred to Eppendorf tubes, centrifuged, and suspended in 200 μl 10mM Tris-HCL, 1mM EDTA (pH 6.8) buffer. Then the suspension was heated in a heating block at 95°C for 20 minutes followed by sonication in an ultrasonic water bath for 15 minutes and then centrifuged at 14000 rpm for 1 minute. Finally, the supernatant was stored at -20°C until used. A species identification was performed as described previously [7].

### 24-loci MIRU-VNTR typing and spoligotyping

The DNA lysates from heat inactivated liquid cultures were shipped to the Research Center Borstel, Germany. All isolates confirmed as *Mycobactrium tuberculosis* using the GenoType MTBC assay (Hain Life science GmbH, Nehren, Germany) were further analyzed using 24 locus mycobacterial interspersed repetitive units-variable number of tandem repeats (MIRU-VNTR) typing and spoligotyping according to the standardized protocols [11–13]. Briefly, MIRU-VNTR alleles were amplified using Quadruplex PCR Kit (Genoscreen, Lille, France) according to the manufactures instructions. Fragment analysis using the GeneScan™ 1200 LIZ® dye as a size standard (Life Technologies, Darmstadt, Germany) was carried out on a capillary sequencer 3130xL and 3500xL for the genetic analyzer. The GeneMapper software v3.7 (Life Technologies, Darmstadt, Germany) was used to determine the copy number of MIRU-VNTR alleles. In the case of one heterogeneous allele call per isolate, we used the higher copy number for the analysis; more than one ambiguous MIRU-VNTR locus per strain was assumed to be the result of a mixed infection and the patient was excluded from the analysis. Molecular typing data were analyzed using BioNumerics v6.7 software (Applied Maths, St. Martens, Belgium) according to the manufacturer’s instructions. For phylogenetic classification of the strains, we used the tree based identification option on the MIRU-VNTRplus website www.miru-vntrplus.org using similarity search option to classify lineages based on the best match with the reference strains in the data base [14, 15]. In addition, the MLVA 15–9 nomenclature type was assigned for each isolate. Lineage 7 (i.e. Ethiopia 1) isolates were defined by a deletion of spacer 4–24 as recently reported by Firdessa et al in 2013 [16]. A cluster was defined as two or more isolates harboring identical MIRU-VNTR and spoligotype profiles. As a surrogate marker for strains associated to a recent chain of transmission, the clustering rate was calculated as strains in cluster/all strains. A dendrogram was generated using the unweighted pair group method with arithmetic means (UPGMA), the minimum spanning tree algorithm used by the Bionumerics software was utilized to analyze the relationship of all samples based on their 24-loci MIRU-VNTR profile. The neighbor joining tree algorithm was applied to set the Ethiopian TBLN isolates into a phylogenetic perspective together with the MTBC reference collection hosted on miru-vntrplus.org.

### Statistical analysis

All data were entered, cleared, and analyzed using the SPSS statistical software package, Vr16 (SPSS Inc., Chicago, IL, USA). Logistic regression model was performed to assess variables associated with clustering in terms of the odds ratio and its 95% confidence interval (CI). The chi-squared test was applied to compare categorical data. A p-value less than or equal to 0.05 was considered significant.

### Ethical clearance

The study was reviewed and approved by an Institutional Review Board (IRB) of the University of Bahir Dar, Bahir Dar, Ethiopia. After the research staff explained about TBLN, the need for screening, the benefits of receiving treatment for prevention and control of TB in the community, written informed consent was obtained from each study subject. Individual records were coded and accessed only by research staff.

## Results

### Study population and diversity of lineages

**Fig 1:**
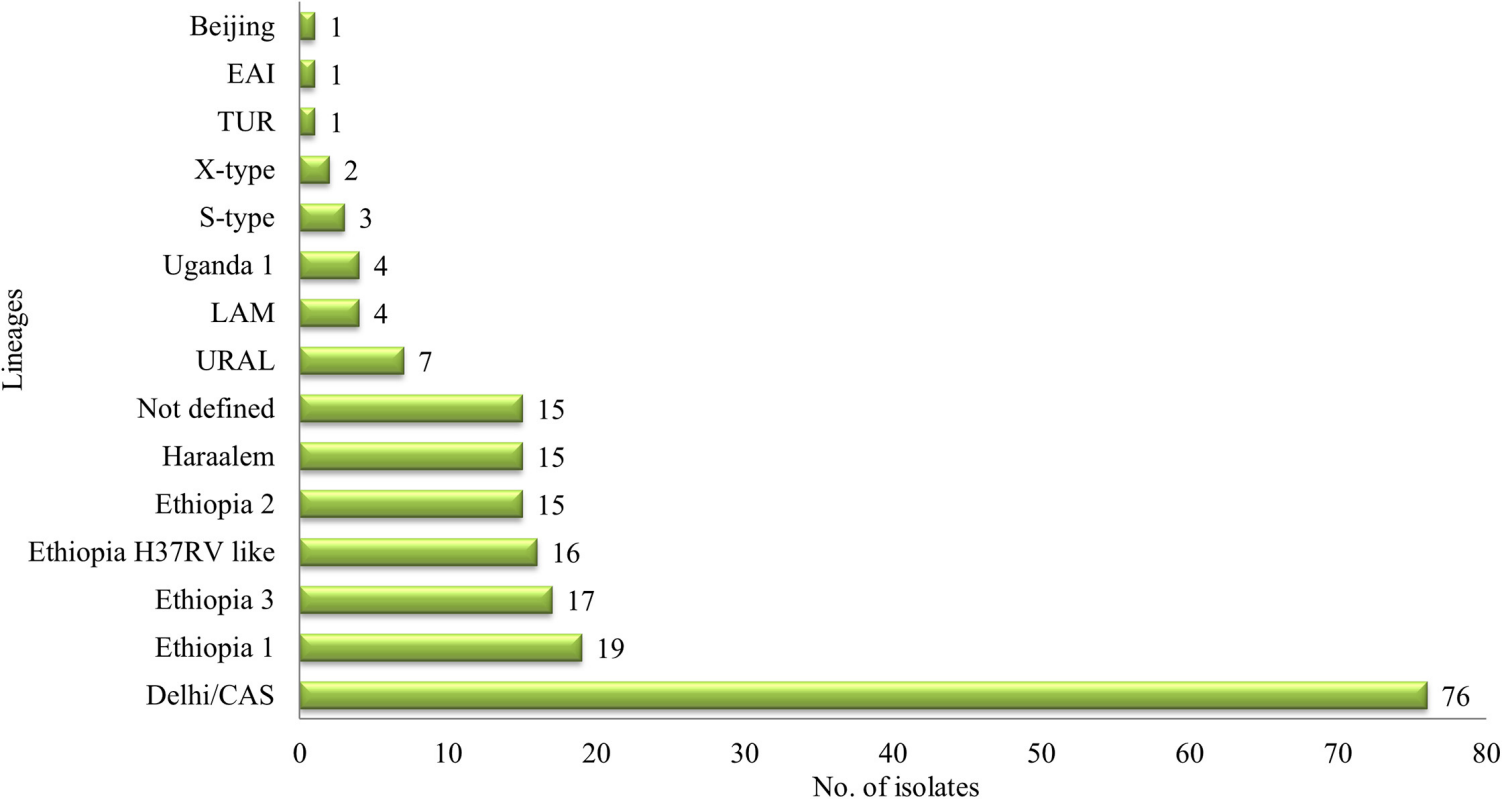
Diversity of lineages among study subjects (N = 196)*.

**Fig 2:**
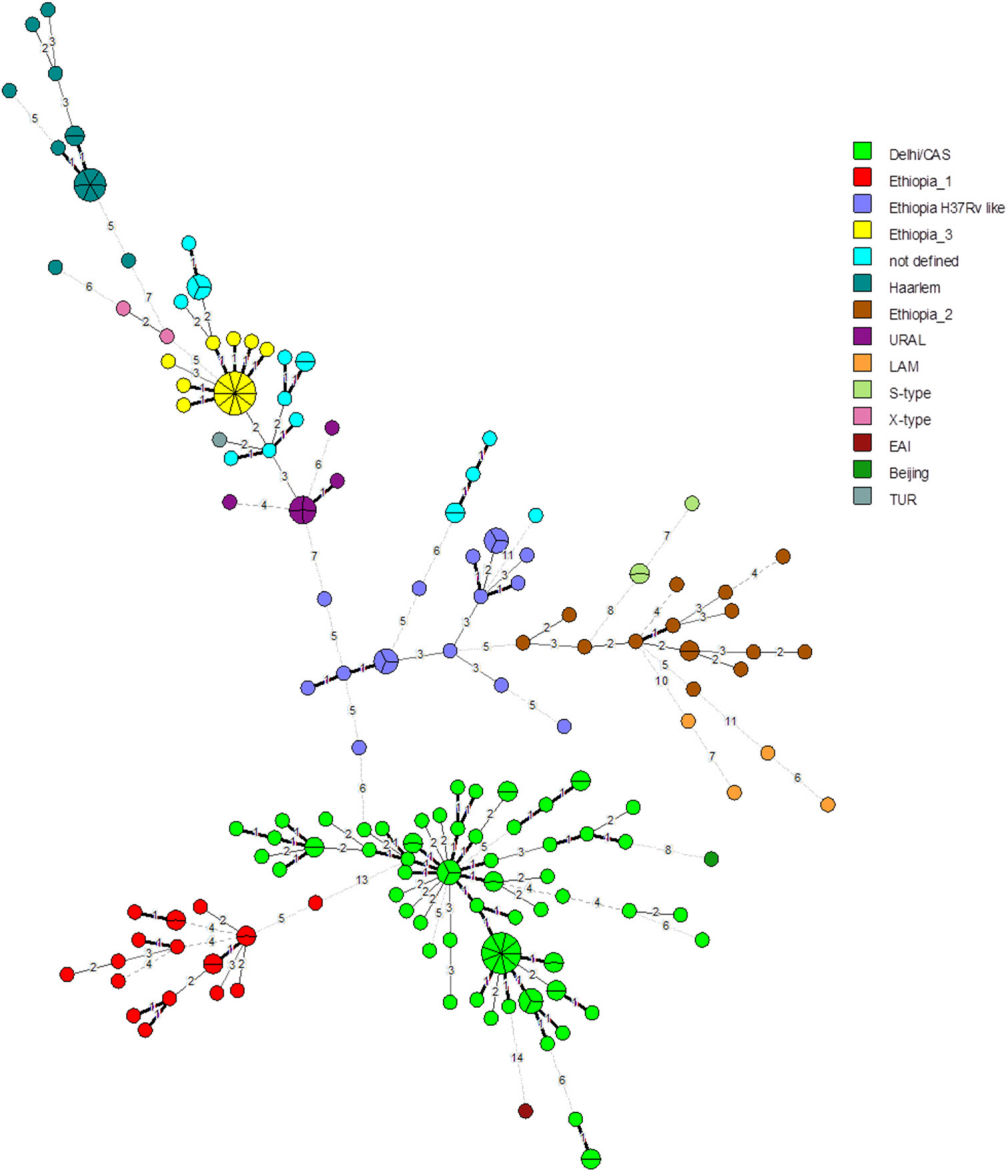
Minimum spanning tree (MST) based on 24-loci MIRU-VNTR data of 196 strains isolated from TBLN cases from Ethiopia. Node size represents number of strains with identical MIRU-VNTR profiles and number given for each branch reflect the number of different MIRU-VNTR alleles between nodes. Identified genotypes are color coded.

DNA extraction was carried out for a total of 226 *Mycobactrium tuberculosis* strains, isolated from TBLN patients. Thirty isolates did not showed sufficient DNA recovery to provide adequate PCR products for the 24-loci MIRU-VNTR analysis and were excluded. However, 196 isolates were employed into a combined 24-locus MIRU-VNTR and spoligotyping analysis. Among the TBLN patients, the *Mycobactrium tuberculosis* population structure was found highly diverse and comprised 14 different genotypes: 76 (38.8%) of the analyzed isolates belonged to the Delhi/CAS lineage, 19 (9.7%) to Ethiopia 1, 17 (8.7%) to Ethiopia 3, 16 (8.2%) to a clade termed Ethiopia H37Rv like, 15 (7.7% each) to Ethiopia 2 and Haarlem, 7 (3.6%) to URAL, Ugandal and LAM comprised 4 (2% each) strains, 3 (1.5%) strains were assigned to S-type, 2 (1%) strains belonged to X-type, while the TUR, EAI, and Beijing were each individually represented in 1 (0.5%) of the patients. Fifteen (7.7%) isolates did not match to known phylogenetic MIRU-VNTR lineages using the MIRU-VNTR plus database and were named as “not defined” in this study (Fig 1 and S1 Fig). The recently defined subgroups (genotypes) Ethiopian 1–3 all have characteristic spoligotyping profiles, that is the lack of spacer 4–24 (Ethiopia 1, i.e. lineage 7), lack of spacer 13 (Ethiopia 2) and lack of spacer 10–19 (Ethiopia 3) (S1 Fig). MIRU-VNTR based phylogenetic classifications also allowed the identification of an Ethiopia H37Rv like clade (Fig 2 and S1 Fig). In comparison to the reference collection on miru-vntrplus.org we can confirm the distinct phylogenetic origin of Ethiopia 1 with its intermediate position between ancestral MTBC strains (e.g. *M*. *africanum*) and modern MTBC strains based on 24-loci MIRU-VNTR profiles (S2 Fig). The proportions of identified genotypes are comparable with the data obtained by Tessema et al. in 2013[17] (Table 1).

**Table 1:** Distribution of defined and non-defined clades of TB in northern Ethiopia among TBLN and PTB cases in two different studies.

Lineages	Present study (n = 196 TBLN cases)	Belay *et al* (17) (n = 244 PTB cases)	p-value
Delhi/CAS	76(38.8)	95(38.9)	0.97
Haarlem	15(7.8)	21(8.6)	0.85
URAL	7(3.6)	8(3.3)	0.84
Ethiopia 1	19(9.7)	19(7.8)	0.59
Ethiopia 2	15(7.8)	9(3.9)	0.12
Ethiopia 3	17(8.7)	32(13.1)	0.19
Ethiopia H37Rv-like	16(8.2)	17(8.8)	0.77

All lineages, except TUR, EAI, and Beijing, were found in both urban and rural residents. However, the strains from the Delhi/CAS lineage were found predominantly (p-value = 0.05) in rural areas. The overall rate of any drug resistance in this study was found to be 6.6% (13/196). Out of the 3 (1.5%) MDR-TB cases identified in this study, two strains belonged to Delhi/CAS and one strain was classified as LAM genotype. Two of the MDR cases were found to be clustered. Strains of the Delhi/CAS genotype were associated with drug resistance (p-value = 0.02). We also analyzed the proportions of different factors considering strains from the Euro-American lineages and the geographically confined clade Ethiopia 1 and we found no factors that are significantly associated to these clades (Table 2).

**Table 2:** Factors associated with identified MTBC clades in Ethiopia (N = 196).

Variables	Lineages*							
	Delhi/CAS (n = 76)	p-value	EA# (n = 84)	p-value	Ethiopia 1 (n = 19)	p-value	ND (n = 15)	p-value
Sex		0.78		0.89		0.53		0.88
Male	30(39.5)		30 (35.7)		7(36.8		6(40)	
Female	46(60.5)		54(64.3)		12(63.2)		9(60)	
Age		0.99		0.36		0.77		0.98
0–14	8(10.5)		7(8.3)		4(21.1)		2(13.3)	
15–29	38(50)		42(50)		9(47.4)		7(46.7)	
30–44	19(25)		22(26.2)		5(26.3)		4(26.7)	
≤ 45	11(14.5)		13(15.5)		1(5.3)		2(13.3)	
Residence		0.05		0.15		0.93		0.14
Urban	29(38.2)		25(29.8)		3(15.8)		2(13.3)	
Rural	47(61.8)		59(70.2)		16(84.2)		13(86.7)	
Sampling site		0.19		0.99		0.15		0.34
Bahir Dar®	50 (65.8)		66(78.6)		14(73.4)		12(80)	
Dessie	15(19.7)		8(9.5)		3(15.8)		3(20)	
Gondar	11(14.5)		10(11.9)		2(10.5)		0	
DST profile		0.02		0.22		0.36		0.28
Susceptible§	67(88.2)		80(95.2)		19(100)		15(100)	
Resistantμ	9(11.8)		4(4.8)		0		0	
Rx history		0.27		0.33		0.28		0.33
New	70(92.1)		81(96.4)		17(89.5)		15(100)	
Retreated	6(7.9)		3(3.6)		2(10.5)		0	
LN region		0.50		0.89		0.46		0.67
Cervical	51 (67.1)		65(77.4)		13(68.4)		12(80)	
Axilla	16(21.1)		11(13.1)		4(21.1)		3(20)	
Inguinal	7(9.2)		6(7.1)		1(5.3)		0	
Other	1(1.3)		2(2.4)		0		0	
LN clinical feature		0.78		0.86		0.98		0.35
Matted	54(71.1)		64(76.2)		14(73.7)		14(93.3)	
Discrete	16(21.1)		17(20.2)		3(15.8)		1(6.7)	
Other	1(1.3)		1(1.2)		0		0	

DST = drug susceptibility test, ND, not defined

# EA, Euro American and it includes LAM, Haarlem, S-type, X-type, TUR, URAL, Ugandal, Ethiopia 2–3 and Ethiopia H37Rv-like strains

®includes Felege Hiwot and Gamby hospitals

§ = susceptible to all anti TB drugs

μ = resistant to at least one anti-TB drug

*the isolated lineages are named according to the database on www.miru-vntrplus.org

### Molecular clustering rate analysis

The cluster analysis, for which a cluster was defined as a minimum of two strains exhibiting identical genotyping patterns (MIRU-VNTR and spoligotype) showed an overall cluster rate of 35% (68/196) including 23 clusters composed of 2–10 strains. The largest clusters were observed for strains belonging to the Ethiopia 3 lineage (MLVA type 594–15, 10 isolates), and Delhi/CAS lineage (MLVA type 1061–32, 8 isolates). The strain clustering rate varied between dominating lineages, however, it was found highest for Ethiopia 3 with 58.8% (10/17) compared to Delhi/CAS 39.5% (30/76), Ethiopia 1 21.1% (4/19), Ethiopia H37Rv-like 31.2% (5/16), Ethiopia 2 13.3% (2/15), and Haarlem 33.3% (5/15) (Table 3, Fig 2 and S1 Fig).

**Table 3:** Phylogenetic diversity of *M*. *tuberculosis* strains within clusters.

Lineages*	TB lymphadenitis						
	Total isolates	Strains-unique	Strains- cluster	No. of cluster	C (%)	Cluster size	MIRU-VNTR 24 loci profile
Delhi/CAS#	76	46	30	11	39.5	2	226425153433324244223374
						3	226425153533324244223374
						2	226425163633124244223374
						8	226425153633124244223374
						2	226425123633123244223374
						2	226425153533322264223384
						2	226425153532322264223384
						2	226325143533324242223374
						2	227425113434423244223254
						2	226425153531324244223374
						3	226425153634124244223374
EthioH37Rv-like	16	11	5	2	31.3	3	223125143324342334223452
						2	223125143325242333223432
Ethiopia 2	15	13	2	1	13.3	2	222325153321243233223362
Ethiopia 3	17	7	10	1	58.8	10	225125113322143134423373
Ethiopia 1	19	15	4	2	21.1	2	147424263663444433234183
						2	148424273573444433244183
ND	15	11	4	2	26.7	2	225125113323143234423383
						2	245125113322043234423393
Haarlem	15	10	5	1	33.3	5	225313153323232632323372
URAL	7	3	4	1	57.1	4	228225113322343234423393
S-type	3	1	2	1	66.7	2	232315123323341444223352
Ugandal	4	2	2	1	50	2	223225152224253334223452
Total	187	119	68	23	35		

#Two MDR TB Delhi/CAS strains in cluster, ND, not defined, C, clustering rate, MLVA, multi-locus variable number tandem repeat analysis

*the isolated lineages are named according to the database on www.miru-vntrplus.org

### Factors associated with clustering

In the multivariate logistic regression model, resistance to any anti-TB drug was associated with the risk of belonging to a TB cluster, vice versa the infection with a susceptible isolate was found to have a reduced risk belonging to a TB transmission chain (i.e. a clustered case) [95% confidence interval (CI), 0.01–0.37]. The same is true for the patients’ age group 15–29 years [95%CI, 0.12–0.87] that had a lower risk to be identified as “recently infected” compared to the age group >45 years (Table 4). Although there was no statistical significance, a higher clustering rate was observed in newly treated patients (35.1%) compared to retreated cases (27.3%) and in rural (35.8%) than urban residents (32.2%). Moreover, sex, lymph node region, sampling area, strain lineages like Delhi/CAS, Haaralem, Ethiopia 1, Ethiopia 2, Ethiopia 3, Ethiopia H37Rv-like strain, URAL, and the “not defined” did not show an association with clustering (Table 4).

**Table 4:** Phylogenetic lineages clustering among different variables of TBLN patients (N = 196).

Variables	Clustered	Unique	COR(95%CI)	AOR(95%CI)
Sex				
Male	26(34.7)	49(65.3)	0.99(0.55–1.82)	1.41(0.68–2.90)
Female	42(34.7)	79(65.3)	Ref	Ref
Age group				
0–14	8(38.1)	13(61.9)	2.03(0.64–6.49)	0.54(0.15–1.99)
15–29	28(28.6)	70(71.4)	3.13(1.30–7.51)*	0.32(0.12–0. 87)*
30–44	17(34)	33(66)	2.43(0.93–6.33)	0.46(0.15–1.38)
≥ 45	15(55.6)	12(44.4)	Ref	Ref
Residence				
Urban	19(32.2)	40(67.8)	0.85(0.45–1.63)	0.92(0.42–2.05)
Rural	49(35.8)	88(64.2)	Ref	Ref
Drug susceptibility test				
Susceptible§	60(32.8)	123(67.2)	0.31(0.09–0.97)*	0.06(0.01–0.37)*
Resistantμ	8(61.5)	5(38.5)	Ref	Ref
Rx history of patients				
New	65(35.1)	120(64.9)	1.44(0.37–5.63)	3.75(0.43–32.32)
Retreated cases	3(27.3)	8(72.7)	Ref	Ref
Lymph node regions				
Cervical	53(37.1)	90(62.9)	0.85(0.08–9.59)	2.26(0.14–36.95)
Axilla	10(29.4)	24(70.6)	1.20(0.09–14.78)	1.83(0.10–32.79)
Inguinal	4(28.6)	10(71.4)	1.25(0.09–17.98)	1.01(0.05–21.59)
Other	1(33.3)	2(66.7)	Ref	Ref
Sampling site				
Bahir Dar	55(38.5)	88(61.5)	0.70(0.27–1.81)	0.91(0.29–2.85)
Dessie	6(20)	24(80)	1.75(0.49–6.17)	0.19(0.03–1.03)
Gondar	7(30.4)	16(69.6)	Ref	Ref
Strain lineage#				
Delhi/CAS	30(39.5)	46(60.5)	0.56(0.16–1.91)	1.64(0.43–6.31)
Haaralem	5(33.3)	10(66.7)	0.72(0.15–3.49)	1.53(0.29–8.13)
Ethiopia 1	4(21.1)	15(78.9)	1.36(0.28–6.68)	0.91(0.17–4.91)
Ethiopia 2	2(13.3)	13(86.7)	2.36(0.36–15.46)	0.42(0.06–3.04)
Ethiopia 3	10(58.8)	7(41.2)	0.26(0.06–1.14)	2.5(0.50–12.45)
Ethiopia H37Rv-like	5(31.3)	11(68.7)	0.80(0.17–3.79)	1.30(0.25–6.75)
URAL	4(57.1)	3(42.9)	0.27(0.04–1.79)	3.58(0.48–26.49)
Ugnadal	2(50)	2(50)	0.36(0.04–3.52)	2.9(0.25–34.59)
S-type	2(66.7)	1(33.3)	0.18(0.01–2.59)	6.93(0.45–106.7)
Not defined	4(26.7)	11(73.3)	Ref	Ref

§ = susceptible to all anti TB drugs

μ = resistant to at least one anti-TB drug, Rx = treatment, Ref = reference, COR = crude odds ratio, AOR = adjusted odds ratio, CI = confidence interval

# only strain lineages that were distributed in clustered and unique

*statistically significant, p≤ 0.05

## Discussion

With the previous report on PTB samples in northwest Ethiopia [17] and this study on TBLN samples, we have expanded the data on the genetic diversity of *Mycobactrium tuberculosis* isolates from patients in northern (west and east) regions in Ethiopia and confirmed the presence and abundance of the new lineage 7 Ethiopia (i.e. Ethiopia 1) with 24-loci MIRU-VNTR typing. Moreover, the ability of this standardized genotyping technique to accurately describe the regional population structure was demonstrated and has revealed new, not yet well-defined, MTBC clades, e.g. Ethiopia 2, Ethiopia 3, and Ethiopia H37Rv-like. Furthermore, we can confirm the observation from Firdessa et al [16] that the population structure of MTBC strains among TBLN patients resembles the structure observed from PTB and TBLN patient derived isolates in Ethiopia.

In this study, dominating strains found in northern Ethiopia among TBLN cases were associated with the Delhi/CAS genotype in line with other reports using solely spoligotyping data [21–27]. Likewise, studies conducted elsewhere in the world reported rates of the Delhi/CAS lineage ranging from 12–67.7% [22, 24, 26, 27, 28, 29], indicating the successful spread of Delhi/CAS strains across regions through population movement [17, 27, 30, 31]. In the present study, the rate of Delhi/CAS genotype strains was slightly higher among rural residents. At the same time, identical MIRU-VNTR profiles were observed among strains originating from the urban settings. This might represent the transmission of Delhi/CAS genotype strains due to extensive social and/or business relations and related traffic between the two geographical regions [32, 33].

Furthermore, we confirmed the presence of newly defined MIRU-VNTR based clades of *Mycobactrium tuberculosis* strains in Ethiopia, namely Ethiopia 1–3 and Ethiopia H37Rv-like, which was reported in previous reports in Ethiopia in 2013 [17] from PTB cases. The genetic diversity of *Mycobactrium tuberculosis* isolates from TBLN patients in our study was similar compared to the isolates from PTB patients reported previously in the country, reflecting the common source of infection for TBLN and PTB patients [16]. This also indicates the absence of pathogen-specific genetic determinants that enhance the risk of developing a disseminated form of the disease [34].

Considering the spoligotype patterns and the classification of lineage 7 Ethiopia (based on the genome data), reported by Firdessa et al in 2013 [16], we found the MIRU-VNTR lineage Ethiopia 1 matches this classification sharing the characteristic lack of spoligotyping spacer 4–24. The analysis of the population structure based on 24-loci MIRU-VNTR data in comparison to the MTBC reference collection hosted on miru-vntrplus.org confirmed the phylogenetic intermediate state between clade 1 (often termed “ancestral”) and clade 2 (‘modern”) MTBC strains [35, 36]. The MIRU-VNTR lineages Ethiopia 2, Ethiopia 3, and Ethiopia H37Rv-like are clearly distinct from Ethiopia 1 isolates and represent other new local subclades of MTBC strains associated with the clade 1 classification of MTBC strains (S2 Fig).

The clustering rate (strains with identical genotyping pattern) in this study was found to be 35%, which is comparable with the rates reported by others [17, 27, 37, 38]. However, the cluster rate was significantly lower (p = 0.035) than the rate reported in the study of PTB in northwestern Ethiopia (45.1%) [17]. In this study, the clustering rate is high among strains associated to S-type (66.7%), Ethiopia 3 (58.8%), Ugnadal (50%), and Delhi/CAS genotype strains (39.5%). Taking the low numbers for S-type and UgandaI genotype strains into account, Ethiopia 3 and Delhi/CAS strains remain the predominant source of the most recent infection/transmission events for TBLN cases.

The overall rate of MDR-TB can still be considered low and is comparable to adjacent regions [17, 24].The higher risk of Ethiopian cases with any drug resistance belonging to a cluster point out the importance of detection and surveillance of drug resistant TB in local TB treatment programs and needs to be carefully considered to prevent an increase of MDR-TB rates in the region.

Studies have reported that higher cluster rates tend to occur among female TB patients compared to males [38]. However, our findings revealed that female and male patients showed an equal rate of clustering. The age group cluster analysis showed the lowest rate of clustering in the group aged 15–29 years at 28.6%, indicating remote transmission of TB in this young age, which is in contrast with the study conducted by Al-Hajoj et al in 2013 [27].

In conclusion, our study showed that lymph node samples are dominated by Dehli/CAS genotype strains and strains of largely newly defined clades based on MIRU-VNTR 24-loci nomenclature, which should be further characterized using whole genome sequencing to identify possible genetic factors contributing to a putative adapting to local host immune systems and improve the understanding of host-pathogen co-evolution. Overall, we found no indication that particular genotypes are specifically associated with TBLN patients.

## Supporting Information

S1 Fig Relationship of 196 Ethiopian M. tuberculosis TBLN samples clustered by unweighted pair group method with arithmetic mean (UPGMA) based on 24 loci MIRU-VNTR genotyping profiles. Identified genotypes are color coded, spoligotyping profiles, cluster numbers and MLVA 15–9 codes are given for all samples.
(PDF)

S2 Fig Neighbor joining (NJ) phylogenetic tree based on 24 loci MIRU-VNTR profiles including 196 TBLN samples from Ethiopia in relation to the *M*. *tuberculosis* complex (MTBC) reference collection hosted on miru-vntrplus.org. Ethiopian samples are color coded according to their genotype. Ethiopia_1, i.e. lineage 7 (light green) is a phylogenetic clade intermediately located between "modern" MTBC strains, e.g. Delhi/CAS, and "ancestral" MTBC strains, e.g. East African Indian (EAI) and *M*. *africanum* strains.
(PDF)
