# Supplementary material for: Tuberculous Lymphadenitis in Ethiopia Predominantly Caused by Strains Belonging to the Delhi/CAS Lineage and Newly Identified Ethiopian Clades of the Mycobacterium tuberculosis Complex
Source: PLoS One. 2015 Sep 16;10(9):e0137865. doi: 10.1371/journal.pone.0137865 (PMC4573740; doi:10.1371/journal.pone.0137865)
Supplement: S1 Fig — Identified genotypes are color coded, spoligotyping profiles, cluster numbers and MLVA 15–9 codes are given for all samples. (PDF) [file pone.0137865.s001.pdf]

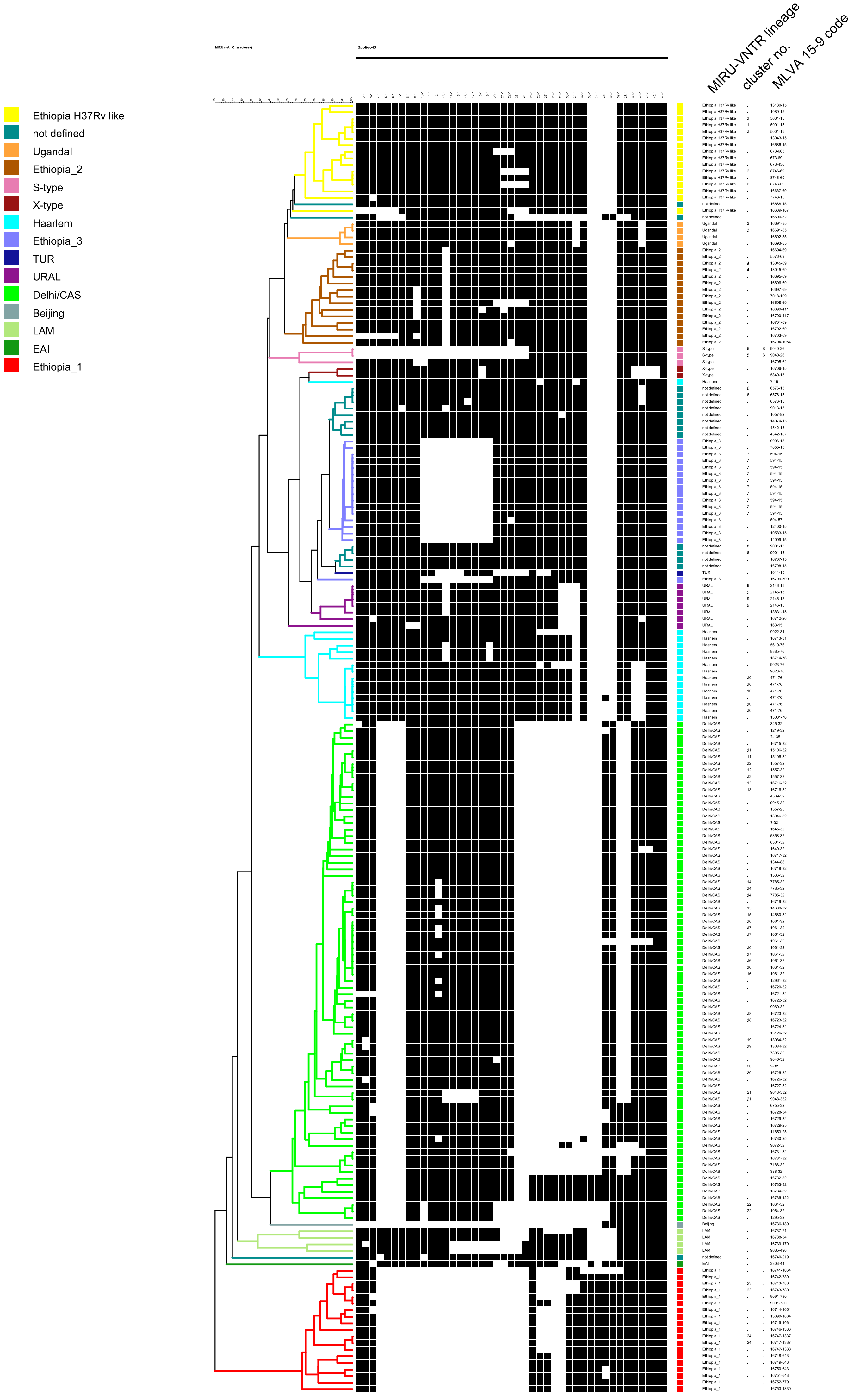

Ethiopia H37Rv like
not defined
Ugandal
Ethiopia_2
S-type
X-type
Haarlem
Ethiopia_3
TUR
URAL
Delhi/CAS
Beijing
LAM
EAI
Ethiopia_1
MIRU (<All Characters>)
Spoligo43
MIRU-VNTR lineage
cluster no.
MLVA 15-9 code
