# Supplementary figures and images for: Tuberculous Lymphadenitis in Ethiopia Predominantly Caused by Strains Belonging to the Delhi/CAS Lineage and Newly Identified Ethiopian Clades of the Mycobacterium tuberculosis Complex
Source: PLoS One. 2015 Sep 16;10(9):e0137865. doi: 10.1371/journal.pone.0137865 (PMC4573740; doi:10.1371/journal.pone.0137865)

NJ-Tree, MIRU-VNTR [24]: Categorical

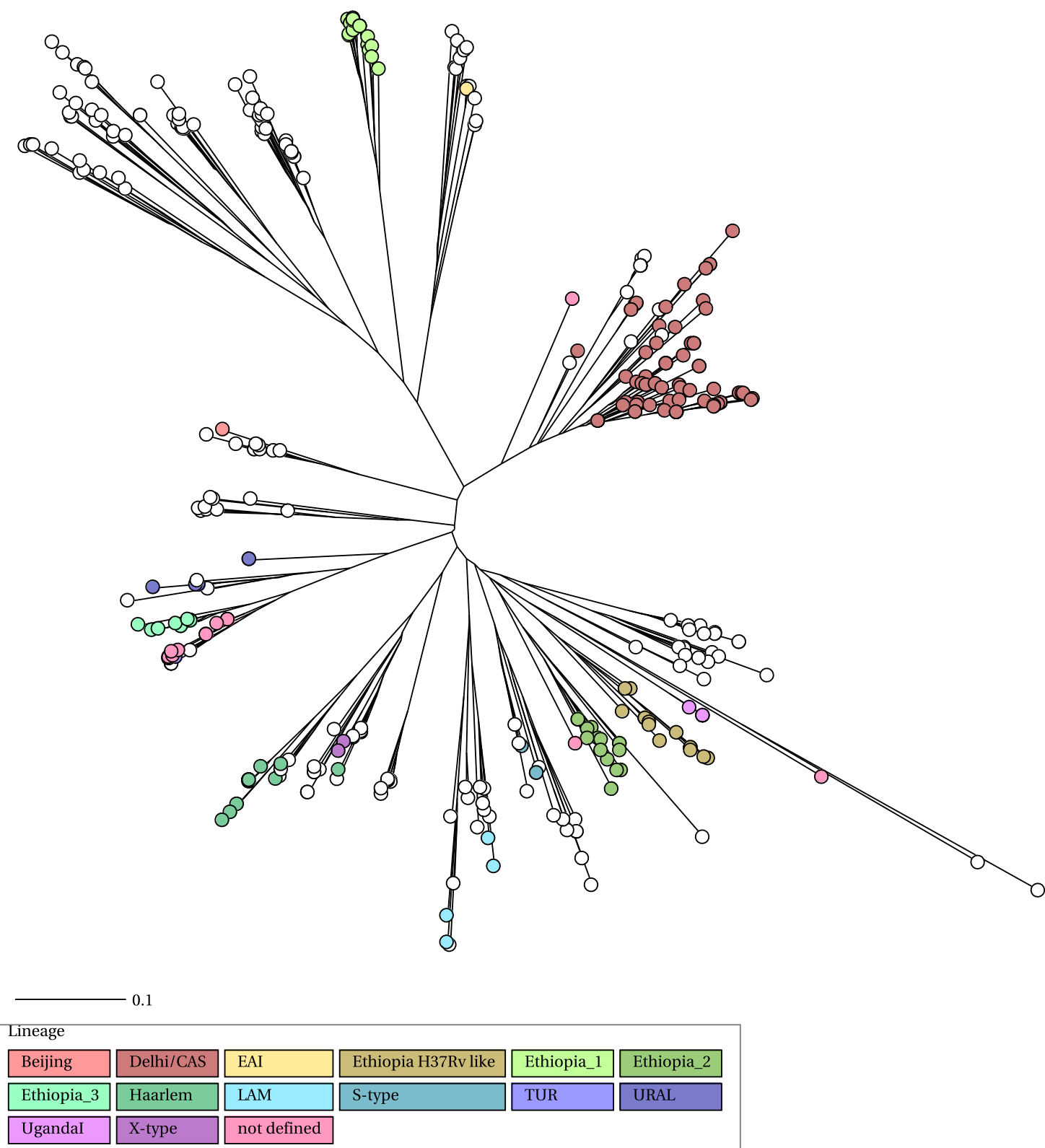

Supplement: S2 Fig — Ethiopian samples are color coded according to their genotype. Ethiopia_1, i.e. lineage 7 (light green) is a phylogenetic clade intermediately located between "modern" MTBC strains, e.g. Delhi/CAS, and "ancestral" MTBC strains, e.g. East African Indian (EAI) and M. africanum strains. (PDF) [file pone.0137865.s002.pdf]
